# Salpingectomy before assisted reproductive technologies: a systematic literature review

**DOI:** 10.1186/s13048-016-0284-1

**Published:** 2016-11-03

**Authors:** Marco Noventa, Salvatore Gizzo, Carlo Saccardi, Shara Borgato, Amerigo Vitagliano, Michela Quaranta, Pietro Litta, Michele Gangemi, Guido Ambrosini, Donato D’Antona, Stefano Palomba

**Affiliations:** 1 Department of Woman and Child Health, University of Padua, Padua, Italy; 2 Department of Obstetrics and Gynecology, NHS Trust - Northampton General Hospital, Northampton, United Kingdom; 3 Unit of Reproductive Surgery and Medicine, ASMN-IRCCS of Reggio Emilia, Reggio Emilia, Italy

**Keywords:** Assisted reproduction, Hydrosalpinx, Infertility, Ovarian reserve, Salpingectomy

## Abstract

Salpingectomy is largely used in case of hydrosalpinx in infertile women scheduled for assisted reproductive technologies (ART), whereas there is no consensus on its role in absence of hydrosalpinx. The current is a systematic literature review to collate all available evidence regarding salpingectomy as fertility enhancement procedure before ART in infertile patients. Our primary endpoint was to assess the impact of the surgical procedure on ovarian reserve, and secondary outcomes were to evaluate its benefits and harms on ART outcomes. We identified 29 papers of which 16 reporting data on the impact of tubal surgery on ovarian reserve and 24 (11 previously included) on ART outcomes. Available data suggested an absence of variation in ovarian reserve markers after unilateral salpingectomy while contradictory results were reported for bilateral surgery. Considering ART outcomes, data reported a significant improvement in ongoing pregnancy/live-birth rate in treated subjects without significant reduction in ovarian response to gonadotropin stimulation. In case of tubal disease, a surgical approach based on unilateral salpingectomy may be considered safe, without negative effects on ovarian reserve and ovarian response to controlled ovarian stimulation whilst having a positive effect on pregnancy rate. Data regarding bilateral salpingectomy and ovarian reserve are conflicting. Further trials are needed to confirm both the benefits of salpingectomy before ART and the safety of bilateral salpingectomy on ovarian reserve, and to clarify the role of uni- or bilateral surgery in case of tubal blockage without hydrosalpinx.

## Background

Outstanding advances in technology have made in vitro fertilization (IVF) an everyday treatment for infertility. The rapid development of IVF and embryo transfer (ET) has seen assisted reproduction proposed as a valid choice for women affected by different type of infertility ranging from aging related problems [1–3] to organic pathologies such as tubal factor [4–6].

Assisted reproduction disregards the physiological role of salpinx during reproduction and has thus generated a minimal or absent interest for tubal diseases in women referred to IVF, a fact confirmed by the paucity of evidences available on this topic if compared with a large amount of literature continuously produced on ovarian response, oocytes/embryo quality and endometrial factor. However, it has been demonstrated that, in patients suffering from hydrosalpinx and tubal factor infertility, the overall success of IVF is lower than expected due to implantation failure, miscarriage and ectopic pregnancy [6, 8].

These findings raised speculations regarding the detrimental role of salpinx fluid by both directly by contaminating the uterine cavity (reduction in embryo implantation) [9–11] and/or indirectly by exerting a toxic effect on the implanted embryo (impairment in embryo development) [12, 13]. This led the United Kingdom’s National institute of Health and Clinical Excellence guidelines (NICE) to recommend laparoscopic salpingectomy before assisted reproductive technologies (ART) in case of signs or suspicions of hydrosalpinx [14]. These Guidelines are also supported by data analyzed in a recent Cochrane review emphasizing a superior pregnancy rate when patients with tubal disease underwent laparoscopic salpingectomy (or at least tubal occlusion) prior to IVF treatments [15].

While salpingectomy before ART is universally recommended in case of evident hydrosalpinx [4, 5, 16, 17], no clear recommendations are available for the management of the wide spectrum of tubal pathologies of varying severity such as slight tubal dilatation (uni- or bilateral), previous tubal abortion and negative tubal patency test without hydrosalpinx. No clear recommendations regarding the surgical management of varying degrees of hydrosalpinx nor of unilateral hydrosalpinx with non-patent contralateral tube are available in the literature. The lack of accepted evidences regarding the potential detrimental effects of salpingectomy on ovarian reserve further complicate the determination of the most appropriate management for tubal disease before ART [18–20].

Based on these considerations, we performed a systematic literature review of all available evidence in order to summarize the actual benefits/harms of salpingectomy before ART in patients with and without hydrosalpinx.

## Methods

A systematic literature search (English and French written literature) was conducted by electronic search of public databases MEDLINE, EMBASE, Science-Direct and the Cochrane library. We collected evidences dating from 1998 till 2015. This systematic review was conducted according to the Preferred Reporting Items for Systematic reviews and Meta-Analysis (PRISMA) guidelines. All longitudinal studies which evaluated ovarian reserve and subsequent ART outcomes following tubal surgery for enhancing fertility (i.e. unilateral and/or bilateral salpingectomy, tubal occlusion, etc.) were identified and analyzed. A manual search of the reference lists of the included studies and review articles was successively performed. All references of the retrieved studies were also reviewed to avoid overlooking relevant publications.

Primary outcome was to evaluate whether tubal surgery may have detrimental effects on ovarian reserve. Serum concentration of follicle stimulating hormone (FSH) and/or anti-Müllerian hormone (AMH) and/or antral follicle count (AFC) were considered as markers of ovarian reserve. Differences in ovarian reserve tests from baseline (pre-surgery) or in comparison with untreated (no surgery) arm were considered and discussed. Secondary outcome was to evaluate potential effects (benefits and harms) of tubal surgery on ovarian response in controlled stimulation cycles.

### Search strategy

The key search terms included: “unilateral/bilateral salpingectomy” [Mesh] and “ovarian reserve” OR “AFC” OR “AMH” OR “FSH” OR “ART outcome” OR “IVF outcome”.

The selection of included studies was based on the availability of baseline AFC and/or baseline serum concentration of AMH and FSH in patients treated by unilateral/bilateral salpingectomy (laparoscopy/laparotomy) for benign gynecological disease. We also included all studies which reported in detail ART outcomes in patients with a history of unilateral/bilateral salpingectomy (laparoscopy/laparotomy) compared to untreated controls.

Titles and abstracts from electronic searches were scrutinized and reviewed by two authors independently (M.N; S.B) and full manuscripts and relative citation lists were analyzed by a third reviewer (S.G) with the scope of retrieving any omitted articles and selection of manuscripts by application of inclusion criteria.

Available data independently according to the subsequent topics: “Modifications in basal AMH and FSH after salpingectomy”, “ART after salpingectomy”, “ART outcomes following salpingectomy” were analyzed qualitatively.

### Inclusion and exclusion criteria

Studies were considered eligible if they satisfied the following criteria: *i)* longitudinal prospective, randomized trial or retrospective studies; *ii)* study population comprised exclusively of women of reproductive age undergoing unilateral or bilateral salpingectomy for benign gynecological disease, *iii)* presence of a control group either consisting of healthy matched controls or the same subjects prior to surgery; *iv)* ovarian reserve must be described by basal serum value of AMH and/or FSH and/or AFC for the entire sample; *v)* ART outcome must be described by at least one of the following, number of oocytes retrieved and/or number of obtained embryos and/or pregnancy rate. Exclusion criteria were considered: reviews and case report, duplicated data, and longitudinal studies referring to intrauterine insemination.

## Results

**Fig. 1:**
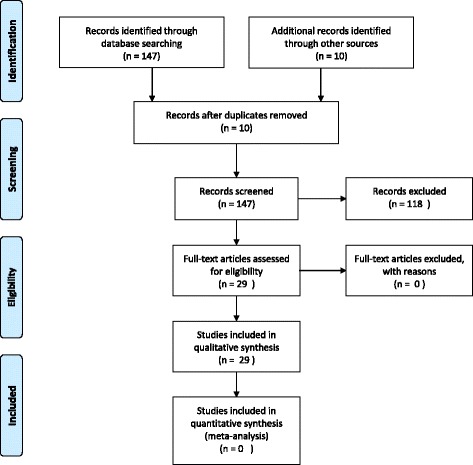
Study’ flow-chart according to PRISMA guidelines

The systematic literature search based on our pre-defined key search items yielded a total of 147 potentially relevant papers. Out of 147 only 29 met the inclusion criteria (Fig. 1) [1–13, 16–31]. A full report regarding authors, study design, sample size, type of surgical procedure (unilateral/bilateral salpingectomy), epidemiological features, surgical indications and years from surgery is illustrated in detail in Table 1.

**Table 1:** Descriptive data of all eligible studies regarding patients, their general features, type and indication for surgery and type/duration of infertilityIncluded

Authors & Years	Type of Study	Patients (total)	Patients (Salpingectomy)	Patients-Controls	Age (year ± SD)	BMI (± SD)	Indication for Surgery	Time from Surgery	Reason of Infertility	Duration of Infertility
Lass et al. [1]	OP	102	a) 29 SLP (ECP) Type: unilateral Surgery: not specified	b) 73 no surgery (healthy subjects)	a) 33.1 ± 4.9 b) 34.2 ± 4.1	*n.r*	a) ECP	2 years	a,b) MF 31; ID 42	2 years after surgery at least
Dechaud et al. [2]	RCT	60	a) 30 SLP (HY) Type: not specified Surgery: LPS	b) 30 diagnostic LPS (TF, HY)	a) 31.7 ± 4.5 b) 30.6 ± 3.3	*n.r*	a,b) salpingitis or HY	a) 10.1 ± 7.5 months b) 9.5 ± 7.2 months (LPS diagnostic)	a,b) TF	a) 55.2 ± 33.3 months b) 48 ± 25.4 months
Bredkjaer et al. [3]	R	278	a) 139 SLP (HY) Type: 128 bilateral, 11partial Surgery: not specified	b) 139 no surgery (TF, no HY)	a) 32.6 (22–39) b) 32.9 (24–40)	*n.r*	a) HY	92 pz between 1.5 and 5 years 47 pz 3–6 months	a,b) TF	n.r
Strandell et al. [4]	RCT	204	a) 116 SLP (HY) Type: not specified Surgery: LPS	b) 88 no surgery (TF, HY)	a) 31.8 ± 3.6 b) 31.8 ± 3.7	*n.r*	a,b) HY uni o bilateral	2 months (at least)	a,b) TF	1 month to 2 years
Dar et al. [5]	R	26	a) 26 SLP (ECP) Type : 25 unilateral, 1 bilateral Surgery: LPS	Control: same patients before surgery	a) 31.7 ± 3.6	*n.r*	a) ECP	a period < 3 years between the cycles (before and after surgery)	a) MEF 16; MF 6 AN 1; END 1; ID 2	n.r
Strandell et al. [6]	OP	26	a) 26 SLP (HY) Type: 9 unilateral and 17 bilateral Surgery: LPS	Control: same patients before surgery	a) 32.7 ± 3.6	*n.r*	a) HY	n.r	*n.r*	n.r
Strandell et al. [7]	RCT	185	a) 103 SLP (HY) Type: 40 unilateral, 63 bilateral Surgery: LPS	b) 82 no surgery (TF, HY)	a) 32.8 ± 3.5 b) 32.5 ± 3.8	*n.r*	a) HY	n.r	a,b) TF	n.r
Surrey et al. [8]	R	94	a) 32 SLP (HY) Type: not specified Surgery: LPS	b) 15 PTO (HY) c) 35 no surgery (TF no HY) d) 12 tubal ligation (prior sterilization)	a) 35.1 ± 0.7 b) 35.4 ± 1.0 c) 35.6 ± 0.7 d) 38.2 ± 1.0	*n.r*	a,b) HY c,d) TF	6 months	a,b,c,d) TF	n.r
Tal et al. [12]	OP	78	a) 26 SLP (ECP) Type: unilateral Surgery: 14 LPT, 12 LPS	b) 52 no surgery (healthy subjects)	a) 32.1 ± 4.1 b) 32.0 ± 5.1	*n.r*	a) ECP	1-9 years	*n.r*	n.r
Chan et al. [13]	R	32	32 SLP (ECP) a) 18 unilateral LPS b) 14 unilateral LPT	Controls: non-operated site	a) 34 (31–38) b) 36 (33–44)	a) 20.96 b) 21.44	a,b) ECP	3 months (at least)	*n.r*	n.r
Kontoravdis et al. [9]	RCT	115	a) 50 POT (HY) b) 50 SLP (HY) Type: not specified Surgery: LPS	c) 15 no surgery (HY)	a) 31 ± 4.5 b) 29.8 ± 3.4 c) 34 ± 5.3	*n.r*	a,b) HY unilateral or bilateral	n.r	*n.r*	n.r
Gelbaya et al. [6]	R	168	a) 40 SLP (HY) Type: 16 unilateral, 24 bilateral b) 25 PTD Type: 9 unilateral, 16 bilateral Surgery: LPS	c) 103 no surgery (tubal factor, no HY)	a) 32.8 ± 3.57 b) 33.1 ± 2.71 c) 33.5 ± 3.32	*n.r*	a,b) HY	3 months at least	*All TF plus:* a) MF 2/40; OF 4/40; END 2/40 b) MF 5/25;OF 5/25; END 1/25 c) MF 12/103; OF 9/103; END 5/103	n.r
Moshin & Hotineanu [11]	RCT	204	a) 60 SLP (HY) b) 78 PTO (HY) Type: not specified Surgery: not specified	c) 66 no surgery (HY)	n.r	*n.r*	a,b) HY	n.r	*n.r*	n.r
Sezik et al. [16]	RCT	24	a) 12 total hysterectomy + bilateral- SLP Surgery: LPT	b) 12 total hysterectomy without SLP	a) 41.6 ± 1.7 b) 41.1 ± 1.4	a) 24.5 ± 2.2 b) 26.6 ± 4.8	n.r	n.r	*n.r*	n.r
Nakagawa et al. [17]	P	17	a) 6 SLP (HY) Type: not specified Surgery: LPS	b) 11 PTD (HY)	a) 31.7 ± 6.3 b) 35.3 ± 3.6	*n.r*	a,b) HY	n.r	*n.r*	n.r
Orvieto et al. [18]	R	15	a) 15 SLP (HY) Type: not specified Surgery: not specified	Controls: same patients before surgery	a) 32 ± 4.4	24 ± 5.5	a) HY	n.r	*n.r*	n.r
Almog et al. [19]	R	36	a) 36 SLP Type :22 unilateral, 14 bilateral Surgery: LPS	Controls: same patients before surgery	a) 34.2 ± 4.5	*n.r*	a) ECP 21 HY 14 Both 1	152 ± 36 days	*n.r*	n.r
Xi et al. [20]	R	156	a) 76 SLP (ECP) Type: 32 bilateral, 44 unilateral (23 controlateral ligation) Surgery: LPS	Controls: same patients before surgery b) 80 no surgery (healthy subjects)	a) 31.5 ± 4.2	*n.r*	a) ECP	At least 3 months after salpingectomy	a) TF 54 MF 7 END 5	n.r
Na et al. [21]	R	97	a) 41 SLP (HY) Type: not specified Surgery: LPS	b) 56 sclerotherapy (HY)	a) 32.4 ± 4.5 b) 32.9 ± 4.1	a) 22.2 ± 5.0 b) 21.5 ± 2.2	a, b) HY	n.r	a,b) HY	a) 3.8 ± 3.4 b) 2.9 ± 1.8
Ni et al.[22]	PC	134	60 SLP a) 26 bilateral b) 34 unilateral c) 23 PTO Surgery: LPS	d) 51 no surgery (TF, no HY)	a) 29.23 ± 2.98 b) 30.12 ± 3.73 c) 30.65 ± 3.32 d) 29.18 ± 3.36	a) 21.21 ± 2.05 b) 21.37 ± 1.89 c) 20.78 ± 2.04 d) 20.95 ± 1.66	a, b) ECP, HY c) HY	n.r	TF n.s	a) 2.00 ± 1.67 years b) 3.14 ± 2.12 c) 4.61 ± 2.81 d) 3.98 ± 2.44
Uyar et al. [23]	OP	162	a) 33 patients (ECP) - 26 unilateral SLP - 3 salpingostomy - 1 tubal milkink - 1 fimbriectomy - 2 tubal abortion surgery: LPS/LPT	b) 49 MTX (ECP) c) 80 no surgery	a) 31.1 ± 5.1 b) 29.7 ± 5.0 c) 28.9 ± 6.0	*n.r*	a, b) ECP	n.r	*n.r*	n.r
Lin et al. [24]	R	288 cycles in 251 women	a) 103 cycles in 96 SLP Type: not specified Surgery: LPS	b) 185 cycles in 155 women (prior sterilization, tuboplasty, PTO) TF infertility	a) 33.2 ± 4.2 b) 32.8 ± 4.6	a) 22.1 ± 4.3 b) 21.9 ± 3.2	a) ECP or HY	n.a	a,b) TF	n.a
Grynnerup et al. [25]	P-CS	71	a) 16 SLP (HY) Type: (uni/ bilateral) not specified Surgery: LPS	b) 42 no surgery (TF, with or without HY) c) 13 no surgery (unexplained infertility, no HY)	a) 34 (25–37) b) 33 (26–37) c) 32 (27–36)	*n.r*	a) HY	At least 2 months	a) HY 16 b) TF 42 c) ID 13	a) 5 years b) 4 years c) 5 years
Findley et al. [26]	RCT	30	a) 15 hysterectomy + bilateral SLP Surgery: LPS	b) 15 hysterectomy with no SLP LPS	a) 36.6 ± 4.5 b) 37.8 ± 5.0	a) 34.4 ± 6.8 b) 38.1 ± 10.7	a,b) benign indications	n.r	*n.r*	n.r
Hill et al. [27]	R	189	a) 36 SLP (ECP) Type: unilateral Surgery: not specified	Controls: same patients before surgery b) 153 MTX (ECP)	a) 35.8 ± 4.3 b) 34.3 ± 4.5	*n.r*	a,b) ECP	n.r	*n.r*	n.r
Ye et al. [28]	R	198	124 SLP (HY, ECP, TOA) a) 83 unilateral b) 41 bilateral Surgery: not specified	c) 74 no surgery (infertility, no TF)	a) 33,02 ± 4,66 b) 33,58 ± 3,95 c) 33,8 ± 4,67	a) 21,63 ± 2,46 b) 21,1 ± 2,85 c) 21,43 ± 2,83	a) ECP 79 HY 3 TOA 1 b) ECP 24 HY 16 TOA 1	n.r	a) MF 45; MF & FF 28 b) MF 16 MF & FF 15 c) MF 38 MF & FF 26 UNKNOWN 2	a) 0.31 ± 1.13 PI 2.85 ± 2.81 SI b) 0.82 ± 1.96 PI 3.23 ± 3.24 SI c) 3.6 ± 4.15 PI 2.36 ± 3.32 SI
Pereira et al. [29]	R	144	a) 37 SLP (ECP) Type: unilateral Surgery: LPS b) 107 MTX (ECP)	Controls: same patients before surgery or MTX	a) 36.4 ± 3.03 b) 37.1 ± 4.01	a) 24 ± 3.65 b) 23.2 ± 4.23	a,b) ECP	12 months	a) A 31.8 %; TF 3 13.6 %; END 9 %; MF 18.1 %; ID 4.55 %; Other 18.1 % b) AN 32.9 %;TF 12.5 %: END 7.95 %; MF 23.9 % ID 5.68 % Other 15.9 %	n.r
Odesjo et al. [30]	R	153	a) 118 SLP (ECP) Type: unilateral Surgery: not specified	b) 35 unilateral salpingotomy (ECP)	a) 32.5 ± 3.93 b) 33.8 ± 3.07	a) 24.9 ± 4.5 b) 24 ± 3.76	a,b) ECP	a) 3.11 ± 2.90 years b) 6.85 ± 5.05 years	a) TF 92 (78 %) a) other reasons 26 (22 %) b) TF 24 (70.6 %) b) other reason 10 (29.4 %)	n.r
Venturella et al. [31]	RCT	186	a) 91 SLP standard b) 95 SLP wide* Type: bilateral surgery: LPS	Controls: same patients before surgery	a) 41.16 ± 5.33 b) 41.56 ± 5.45	*n.r*	a,b) myomectomy, tubal surgical sterilization	n.a	*n.a*	n.a

*SLP*, salpingectomy; n.r, not reported; n.a, not applicable; ECP, ectopic pregnancy; HY, Hydrosalpinx; TOA, tubo-ovarian abscess; FF, female factor (n.s), TF, tubal factor; OF, ovarian factor; MF, male factor; END, Endometriosis; MEF, mechanical factor; AN, anovulation; ID, idiopathic; R, retrospective; OP, observational-prospective; RCT, randomized controlled trial; P-CS, prospective cross-sectional study; PC, prospective cohort study; LPS, laparoscopy; LPT, laparotomy; PTD, proximal tubal division; PTO, proximal tubal occlusion/ligation; MTX, methotrexate; PI, primary infertility; SI, secondary infertility

The majority of patients underwent a laparoscopic surgical procedure (Table 1). The surgical indication was either hydrosalpinx or ectopic pregnancy in nearly all patients considered; only one manuscript included patients with salpingectomy due to tube-ovarian abscess [28]. Descriptive reports regarding baseline serum FSH and AMH serum values and AFC of patients included in the review are described in detail in Table 2. Descriptive reports concerning ART outcome [in particular: estradiol (E_2_) at ovulation induction, stimulation length, number of oocytes retrieved, obtained embryos, transferred embryos, implantation rate, clinical pregnancy rate, ongoing pregnancy rate] are reported in detail in Table 3.

**Table 2:** Ovarian reserve test (AMH and AFC) and basal-FSH of patients included in the review

OVARIAN RESERVE							
Authors & Years	Patients	AMH ± SD	*p*	AFC ± SD/range	*p*	FSH ± SD/range	*p*
Surrey et al. [8]	a) 32 SLP (HY) Type: not specified b) 15 PTO (HY) c) 35 no surgery (TF no HY) d) 12 tubal ligation	*n.r.*		*n.r.*		a) 7.07 ± 0.12 b) 6.65 ± 0.42 c) 7.08 ± 0.23 d) 7.47 ± 0.25	*ns*
Chan et al. [13]	32 SLP (ECP) a) 18 unilateral LPS b) 14 unilateral LPT	*n.r*	-	*Operated site* a) 5. 0 (3.0-7.3) b) 6.5 (1.8-10.3) *Non operated site* a) 7.5 (4.8-8.3) b) 4.0 (2.8-9.3)	a) *.05* b) *ns*	*n.r*	-
Gelbaya et al. [10]	a) 40 SLP (HY) Type: 16 unilateral, 24 bilateral b) 25 PTD Type: 9 unilateral, 16 bilateral c) 103 no surgery (tubal factor, no HY)	*n.r*	*-*	*n.r*	-	*IU/L* a) 6.8 ± 1.3 pre 7.61 ± 2.31 post b) 6.4 ± 1.5 pre 6.35 ± 1.51 post c) 6.6 ± 2.3 pre 6.71 ± 2.32 post	a) *.05* b) *ns* c) *ns*
Sezik et al. [16]	a) 12 total hysterectomy + bilateral SLP b) 12 total hysterectomy without SLP	*n.r*	*-*	*n.r*	-	a) Basal 4.8 ± 1.4 1 month 4.2 ± 1.6 6 months 4.4 ± 2.1 b) Basal 5.9 ± 1.6 1 month 5.2 ± 1.6 6 months 5.5 ± 1.2	*ns*
Nakagawa et al. [17]	a) 6 SLP (HY) Type: not specified b) 11 PTD (HY)	*n.r*	*-*	*n.r*	-	*IU/L* *Before surgery* a) 8.0 ± 2.9 b) 6.8 ± 1.1 *After surgery* a) 8.6 ± 4.0 b) 14.1 ± 9.3	a) *ns* b) *ns*
Orvieto et al. [18]	a) 15 uni/bilateral SLP (HY)	*n.r*	-	*Before surgery* a) 5.6 ± 2.5 affected side 11.4 ± 4.5 both side *After surgery* a) 4.7 ± 2.3 affected side 9.5 ± 4.9 both side	a) *.05*	*n.r*	-
Xi et al. [20]	a) 76 SLP (ECP) - 44 unilateral - 32 bilateral b) 80 no surgery (healthy subjects)	*n.r*	*-*	*n.r*	-	*IU/L* *Before surgery* a) 6.9 ± 1.5 7.5 ± 1.5 7.0 ± 1.5 *After surgery* a) 7.2 ± 1.6 7.3 ± 1.2 7.2 ± 1.4 b) 7.2 ± 1.8	a) *ns*
Na et al. [21]	a) 41 SLP (HY) Type: not specified b) 56 sclerotherapy (HY)	*n.r*	*-*	*n.r*	-	*IU/L* a) 8.2 ± 4.6 b) 10.9 ± 17.1	*ns*
Grynnerup et al. [25]	a) 16 SLP (HY) Type: not specified b) 42 no surgery (TF, with or without HY) c) 13 no surgery (unexplained infertility, no HY)	*pmol/L* a) 16,1 (5,2-54) b) 23,4 (3,5-50) c) 21.8 (12–64)	a) *ns* b) *ns* c) *ns*	*n.r*	*-*	*n.r*	*-*
Uyar et al. [23]	a) 33 patients (ECP) - 26 unilateral SLP - 3 salpingostomy b) 49 MTX (ECP) c) 80 no surgery	n.r	*-*	a) 10.1 ± 3.5 b) 10.3 ± 4.1 c) 10.1 ± 3.5	a) *ns* b) *ns* c) *ns*	*IU/L* a) 7.7 ± 3.8 b) 7.7 ± 2.3 c) 7.9 ± 1.9	a) *ns* b) *ns* c) *ns*
Ni et al. [22]	60 SLP (ECP, HY) a) 26 bilateral b) 34 unilateral c) 23 PTO (HY) d) 51 no surgery (TF, no HY)	*pg/mL* a) 90.00 b) 100.00 c) 100.00 d) 110.00	a) *ns* b) *ns* c) *ns* d) *ns*	a) 9 b) 10 c) 11 d) 11	a) *ns* b) *ns* c) *ns* d) *ns*	*IU/L* a) 7.35 ± 1.59 b) 7.17 ± 1.16 c) 7.64 ± 2.10 d) 7.13 ± 1.57	a) *ns* b) *ns* c) *ns* d) *ns*
Findley et al. [26]	a) 15 hysterectomy + bilateral SLP b) 15 hysterectomy with no SLP	a) Basal 2.26 ± 2.72 4–6 weeks 1.03 ± 1.04 3 months 1.86 ± 1.99 b) Basal 2.25 ± 2.57 4–6 weeks 1.25 ± 2.09 3 months 1.82 ± 3.12	*ns*	*n.r*	-	*n.r*	-
Hill et al. [27]	a) 36 SLP (ECP) Type: unilateral b) 153 MTX (ECP)	*n.r*	-	a) 10 (3–50) pre 10 (4–45) post b) 12 (1–53) pre 13 (1–60) post	a) *ns* b) *ns*	*IU/L* a) 7.2 (2.6 -16.0) pre 7.9 (5.1-10.4) post b) 6.9 (2.4-14.2) pre 7.2 (2.3-16.3) post	a) *ns* b) *ns*
Pereira et al. [29]	a) 37 SLP (ECP) Type: unilateral b) 107 MTX (ECP)	*n.r*	*-*	*n.r*	-	*mIU/mL* a) 4.98 ± 2.19 pre 4.87 ± 2.97 post b) 4.81 ± 2.75 pre 4.94 ± 2.05 post	a) *ns* b) *ns*
Ye et al. [28]	124 SLP (HY, ECP, TOA) a) 83 unilateral b) 41 bilateral c) 74 no surgery (infertility, no TF)	fmol/mL a) 167,56 ± 127,03 b) 127,11 ± 93,23 c) 183.48 ± 104,37	*.05*	a) 10,7 ± 3,62 b) 9,58 ± 3,73 c) 11,2 ± 4,16	*ns*	mIU/mL a) 8.42 ± 2.3 b) 9.13 ± 3.2 c) 7.85 ± 2.69	*.05*
Venturella et al. [31]	a) 91 SLP standard b) 95 SLP wide Type: bilateral	*ng/mL* *Before surgery* a) 0.93 ± 1.13 b) 0.86 ± 1.01 *Δ After surgery* a) -0.09 ± 0.24 (Δ) b) -0.07 ± 0.22 (Δ)	a) *ns* b) *ns*	*Before surgery* a) 7.8 ± 4.23 b) 6.82 ± 4.68 *Δ After surgery* a) -0.33 ± 0.73 (Δ) b) -0.26 ± 0.61 (Δ)	a) *ns* b) *ns*	*mIU/mL* *Before surgery* a) 12.9 ± 9.71 b) 12.39 ± 7.88 *After surgery* a) 0.47 ± 0.86 b) 0.37 ± 0.84	a) *ns* b) *ns*

n.r, not reported; ns, not significant; LPS, laparoscopy; LPT, laparotomy; MTX, methotrexate; PTD, proximal tubal division; PTO, Proximal tubal occlusion; ° On 21 patients (23 contro-lateral ligation excluded)

**Table 3:** IVF outcome of patients included in the review

IVF TREATMENT																	
Authors & Years	Patients	E2 (on hCG day) ± SD	*p*	Stimulation Lenght (Days ± SD)	*p*	No. Oocytes retrieved ± SD/range	*p*	No. Obtained Embryos ± SD	*p*	No. transferred Embryos ± SD/range	*p*	Implantation Rate % (N)	*p*	Pregnancy Rate % (N)	*p*	Ongoing pregnancy rate % (N)	*p*
Lass et al. [1]	a) 29 SLP (ECP) Type: unilateral b) 73 no surgery (healthy subjects)	*pmol/L* a) 6.087 ± 2.889 b) 6.635 ± 2735	*ns*	a) 12.3 ± 1.6 b) 13.1 ± 2.0	*ns*	*- General* a) 9.9 ± 5.3 b) 9.9 ± 5.3 c) Ipsilateral ovary 3.8 ± 3.0 Controlateral ovary 6.0 ± 3.6	a, b) ns c) .01	*n.r*	-	a) 2.4 ± 0.5 b) 2.0 ± 0.7	*ns*	*n.r*	-	a) 17.2 (5) b) 13.7 (10)	*ns*	*n.r*	*-*
Dechaud et al. [2]	a) 30 SLP (HY) Type: not specified b) 30 no surgery (TF, HY)	*pmol/L* a) 2.699 b) 1.903	*ns*	*n.r*	-	a) 10.1 ± 5.0 b) 10.5 ± 6.0	*ns*	a) 5.2 ± 3.4 b) 4.8 ± 3.7	*ns*	*n.r*	-	*- First attempts* a) 10.4 (5/48) b) 4.6 (2/43) *- All attempts* a) 13.4 (21/156) b) 8.6 (10/116)	*ns*	- *Per cycle* a) 23.7 (14/59) b) 16.3 (8/49) *- Per oocyte retrieval* a) 31.8 (14/44) b) 23.5 (8/34) *- Per transfer* a) 36.8 (14/38) b) 25 (8/32)	*ns*	a) 34.2 (13/38) b) 18.7 (6/32)	*ns*
Bredkjaer et al. [3]	a) 139 SLP (HY) Type: 128 bilateral, 11 partial b) 139 no surgery (TF, no HY)	*n.r*	*-*	*n.r*	*-*	a) 9.3 b) 9.1	*ns*	*n.r*	*-*	a) 2.1 b) 2.1	*ns*	a) 19 b) 21	*ns*	a) 40.3 (106) b) 40.5 (120)	*ns*	a) 21.7 (57) b) 21.6 (64)	*ns*
Strandell et al. [4]	a) 116 SLP (HY) Type: not specified b) 88 no surgery (TF, HY)	*n.r*	*-*	a) 11.4 ± 2.2 b) 11.6 ± 2.9	*ns*	a) 10.6 ± 5.9 b) 10.6 ± 6.1	*ns*	a) 6.8 ± 4.8 b) 7.0 ± 4.9	*ns*	a) 2.0 ± 0.3 b) 2.0 ± 0.4	*ns*	*- Per transfer cycle* a) 22.8 (47/206) b) 18.8 (28/149) *- Ultrasound visible HY* a) 30 (21/70) b) 16.7 (13/78)	*ns* *.05*	a) 36.6 (41) b) 23.9 (22)	*.05*	a) 28.6 (32) b) 16.3 (15)	*.05*
Dar et al. [5]	a) 26 SLP (ECP) Type: 25 unilateral, 1 bilateral Control: same patients before surgery	*n.r*	-	Before surgery a) 10.81 ± 2.45 After Surgery a) 10.68 ± 2.57	*ns*	Before surgery a) - *Operated site* 6.06 ± 3.85 - N*on operated site* 5.07 ± 3.08 After Surgery a) - *Operated site* 5.31 ± 4.22 - *Non operated site* 4.4 ± 3.68	*ns*	*n.r*	-	Before surgery a) 3.56 ± 0.81 After Surgery a) 3.37 ± 0.8	*ns*	After Surgery a) 23.07	-	After Surgery a) 19.23	*-*	*n.r*	*-*
Strandell et al. [6]	Before surgery a) 26 SLP (general) b) 9 unilateral c) 17 bilateral After Surgery a) 26 SLP (general) b) 9 unilateral c) 17 bilateral	*n.r*	*-*	Before surgery a) 11 ± 2.4 b) 12.2 ± 2.4 c) 10.4 ± 2.3 After Surgery a) 11.2 ± 2.3 b) 12.2 ± 2.4 c) 10.6 ± 2.1	*ns*	Before surgery a) 9.4 ± 5.9 b) 9.1 ± 4.4 c) 9.5 ± 6.7 After Surgery a) 8.7 ± 5.7 b) 7.7 ± 4.6 c) 9.2 ± 6.2	*ns*	Before surgery a) 7.0 ± 5.6 b) 6.4 ± 3.9 c) 7.3 ± 6.5 After Surgery a) 5.9 ± 4.3 b) 5.3 ± 4.3 c) 6.2 ± 4.4	*ns*	*n.r*	*-*	*n.r*	*-*	*n.r*	*-*	*n.r*	*-*
Strandell et al. [7]	a) 103 SLP (HY) Type: 40 unilateral, 63 bilateral b) 82 no surgery (TF, HY)	*n.r*	*-*	a) 11.3 ± 2.1 b) 11.4 ± 2.6	*ns*	a) 10.3 ± 5.4 b) 10.6 ± 5.4	*ns*	a) 6.8 ± 4.1 b) 7.1 ± 4.4	*ns*	a) 2.0 ± 0.3 b) 2.0 ± 0.3	*ns*	*n.r*	*-*	ODD RATIO - Total group 1.7 - US visible HY 2.8 - US visible HY bilateral 6.9	*.05*	*HAZARD R (BR)* - Total group 2.1 - US visible HY 3.8 US visible HY bilateral 6.0	*.05*
Surrey et al. [8]	a) 32 SLP (HY) Type: not specified b) 15 PTO (HY) c) 35 no surgery (TF no HY) d) 12 PTO (prior sterilization)	pg/mL a) 2.555 ± 219 b) 2.366 ± 282 c) 2.925 ± 259 d) 2.479 ± 281	*ns*	a) 9.5 ± 0.2 b) 10.1 ± 0.4 c) 9.8 ± 0.2 d) 9.3 ± 0.3	*ns*	a) 16.2 ± 1.2 b) 14.4 ± 1.8 c) 17.5 ± 1.8 d) 12.2 ± 1.3	*ns*	*n.r*		a) 2.79 ± 0.2 b) 3.5 ± 0.4 c) 3.2 ± 0.2 d) 3.0 ± 0.3	*ns*	*n.r*	*-*	a) 57.1 (16/28) b) 46.7 (7/15) c) 52.9 (18/34) d) 58.3 (7/12)	*ns*	*n.r*	*-*
Tal et al. [12]	a) 26 SLP (ECP) Type: unilateral b) 52 no surgery (healthy subjects)	pmol /L a) 5189 ± 3310 b) 5631 ± 3512	*ns*	a) 11.6 ± 3.1 b) 10.8 ± 2.5	*ns*	a) 8.6 ± 5.3 b) 8.4 ± 4.9	*ns*	a) 5.5 ± 3.4 b) 4.0 ± 2.3	*ns*	*n.r*	*-*	*n.r*	*-*	*n.r*	*-*	*n.r*	*-*
Gelbaya et al. [10]	a) 40 SLP (HY) Type: 16 unilateral, 24 bilateral b) 25 PTD Type: 9 unilateral, 16 bilateral c) 103 no surgery (TF, no HY)	*pmol/L* a) 8558 ± 4337.98 b) 11192.7 ± 4167.3 c) 9512.78 ± 4173.9	*.05*	a) 10.35 ± 1.92 b) 10.28 ± 1.02 c) 10.17 ± 1.37	*ns*	a) 10.23 ± 6.08 b) 13.68 ± 5.17 c) 12.92 ± 8.75	*.05* a vs b	a) 6.78 ± 4.58 b) 8.52 ± 4.75 c) 7.80 ± 5.48	*ns*	*n.r*	*-*	a) 18.2 (10/55) b) 12.8 (5/39) c) 11 (18/163)	*ns*	a) 17.5 (7/40) b) 20 (5/25) c) 16.5 (17/103)	*ns*	a) 17.5 (7/40) b) 16.0 (4) c) 13.6 (14)	*ns*
Moshin & Hotineanu [11]	a) 60 SLP (HY) b) 78 POT (HY) c) 66 no surgery (HY) type: not specified	*n.r*	*-*	*n.r*	*-*	a) 10.4 ± 6.0 b) 10.2 ± 5.7 c) 9.8 ± 5.5	*ns*	a) 7.0 ± 4.7 b) 6.9 ± 4.6 c) 6.8 ± 4.6	*ns*	a) 3.4 ± 1.2 b) 3.4 ± 1.3 c) 3.5 ± 1.3	*ns*	*n.r*	*-*	a) 38 (23/60) b) 40 (31/78) c) 12 (8/66)	*.05* *a,b vs c*	*n.r*	*-*
Kontoravdis et al. [9]	a) 50 POT (HY) b) 50 SLP (HY) c) 15 no surgery (HY) type: not specified	*n.r.*	*-*	a) 12.3 ± 2.4 b) 11.9 ± 2.5 c) 13 ± 1.9	*ns*	a) 11.6 ± 4.9 b) 12.1 ± 5.0 c) 10.9 ± 5.1	*ns*	a) 8.7 ± 3.9 b) 8.53 ± 4.0 c) 7.9 ± 5.1	*ns*	a) 2.6 ± 0.6 b) 2.6 ± 0.6 c) 2.6 ± 0.8	*ns*	a) 19.5 b) 24.8 c) 5.6	*.03 a vs c* *.007 b vs c*	a) 44.4 b) 55.3 c) 14.3	*.04* *a vs c* *.007 b vs c*	a) 37.8 b) 48.9 c) 7.1	*.03* *a vs c* *.004 b vs c*
Nakagawa et al. [17]	a) 11 PTD (HY) b) 6 SLP (HY) type: not specified	*pg/mL* a) 1553 ± 1468 b) 1530 ± 896	*ns*	*n.r*	*-*	a) 5.3 ± 4.7 b) 7.5 ± 5.5	*ns*	*n.r*	*-*	*n.r*	*-*	*n.r*	*-*	a) 45.5 b) 50.0	*ns*	*n.r*	*-*
Almog et al. [19]	Before surgery (HY, ECP) a) 36 SLP (22 unilateral, 14 bilateral) After Surgery a) 36 SLP	*pg/mL* Before surgery a) 1899.9 ± 185 After Surgery a) 1997 ± 231	*ns*	Before surgery a) 10.5 ± 0.6 After Surgery a) 10.4 ± 0.4	*ns*	Before surgery a) 10.2 ± 6.6 After Surgery a) 10.3 ± 7.4	*ns*	*n.r*	*-*	*n.r*	*-*	*n.r*	*-*	*n.r*	*-*	*n.r*	*-*
Orvieto et al. [18]	Before surgery (HY) a) 15 uni/bilateral After surgery b) 15 uni/bilateral	*pg/mL* a) 1,996 ± 885 b) 2,020 ± 981	ns	b) 10.5 ± 1.7 a) 10.8 ± 1.5	ns	a) 11.6 ± 5.9 b) 10.2 ± 6.1	ns	n.r	-	a) 2.7 ± 1.1 b) 2.3 ± 0.7	ns			a) 6.7 (1/15) b) 40 (6/15)	*.05*	n.r	*-*
Xi et al. [20]	Before surgery (ECP) a) 76 SLP - 44 unilateral - 32 bilateral After Surgery a) 76 SLP - 44 unilateral - 32 bilateral b) 80 no surgery (healthy subjects)	*pg/mL* Before surgery a) 2663.5 ± 1246 2446.9 ± 983.8° 2512.5 ± 1119.4 After surgery a) 2783 ± 1281.3 2860.8 ± 1509.7 2585 ± 1216.2 b) 2934.8 ± 1234.9	*ns*	Before surgery a) 10.7 ± 1.5 11.1 ± 1.5 10.7 ± 1.6 After surgery: a) 11.1 ± 1.8 11 ± 1.3 10.8 ± 1.9 b) 11.1 ± 1.8	*ns*	Before surgery a) 11.1 ± 5.4 11.3 ± 5.1 11.9 ± 6.0 After surgery a) 11.6 ± 4.1 11.1 ± 4.3 11.9 ± 5.5 b) 11.5 ± 4.4	*ns*	Before surgery a) 8.3 ± 4.4 7.5 ± 2.9 8.2 ± 4.3 After surgery a) 8.4 ± 3.9 8.4 ± 4.0 8.4 ± 3.4 b) 8.0 ± 3.1	*ns*	*n.r*	*-*	*n.r*	*-*	*n.r*	*-*	*n.r*	*-*
Na et al. [21]	a) 41 SLP b) 56 sclerotherapy type: not specified	*n.r*	*-*	*n.r*	*-*	a) 6.2 ± 1.0 b) 12.1 ± 11	*ns*	*n.r*	*-*	*n.r*	*-*	*n.r*	*-*	a) 40 (17/43) b) 38 (23/60)	*ns*	*n.r*	*-*
Lin et al. [24]	a) 103 SLP (HY, ECP) b) 185 (prior sterilization, tuboplasty, PTO) type: not specified	*pg/mL* a) 2153 ± 1239 b) 2340 ± 1529	*ns*	a) 8.8 ± 1.4 b) 8.8 ± 1.5	*ns*	a) 7.4 ± 3.9 b) 7.6 ± 4.1	*ns*	*n.r*	*-*	a) 2.5 ± 0.8 b) 2.5 ± 0.8	*ns*	a) 21.4 (56/261) b) 21.0 (98/465)	*ns*	a) 53.5 (55/99) b) 43.5 (77/177)	*ns*	a) 30.3 (30/99) b) 25.4 (45/77)	*ns*
Ni et al. [22]	a) 26 bilateral SLP b) 34 unilateral SLP c) 23 PTO d) no surgery (TF, no HY)	*n.r*	*-*	a) 8.15 ± 1.29 b) 8.21 ± 1.27 c) 8.83 ± 1.37 d) 8.18 ± 1.35	*ns*	a) 9.15 ± 3.73 b) 11.59 ± 6.14 c) 10.70 ± 4.92 d) 10.82 ± 4.82	*ns*	a) 6.04 ± 2.85 b) 7.74 ± 4.23 c) 7.22 ± 3.25 d) 7.57 ± 3.74	*ns*	a) 1.96 ± 0.45 b) 2.03 ± 0.39 c) 2.0 ± 0.43 d) 1.96 ± 0.20	*ns*	*a) 51 (26/51)* *b) 30.4 (21/69)* *c) 39.1 (18/46)* *d) 28 (28/100)*	*-*	a) 65.4 (17/26) b) 47.1 (16/34) c) 52.2 (12/23) d) 49 (25/51)	*ns*	LBR a) 61.5 (16/26 b) 41.2 (14/34) c) 52.2 (12/23) d) 45.1 (23/51)	*ns*
Grynnerup et al. [25]	a) 16 SLP (HY) Type: (uni/bilateral) not specified b) 42 no surgery (TF, with or without HY) c) 13 no surgery (unexplained infertility, no HY)	*n.r*	*-*	*n.r*	*-*	a) 7 (3–31 range) b) 12 (3–30) c) 9 (9–38)	*.05* *a vs b*	*n.r*	*-*	a) 2 (1–3) b) 2 (1–2) c) 2 (1–2)	*ns*	*n.r*	*-*	*n.r*	*-*	*n.r*	*-*
Hill et al. [27]	Surgery Group (ECP) a) 36 unilateral SLP - pre-surgery - post-surgery Medical Group (ECP) b) 153 methotrexate - pre-treatment - post-treatment	*n.r*	*-*	*n.r*	*-*	a) - 13 (3–31) - 12 (3–31) b) - 14 (2–36) - 14 (0–35)	*ns*	*n.r*	*-*	*n.r*	*-*	*n.r*	*-*	a) 14 (39) b) 68 (44)	*ns*	a) 13 (33) b) 53 (35)	*ns*
Pereira et al. [29]	a) 107MTX (ECP) → 88 IVF b) 37 unilateral SLP (ECP) → 22 IVF	*n.r*	*-*	a) MTX pre 9.55 ± 1.99 post 9.76 ± 2.33 b) SLP pre 9.63 ± 2.21 post 9.86 ± 1.93	*a) ns* *b) ns*	a) MTX pre 12.4 ± 5.77 post 10.6 ± 5.51 b) SLP pre 12.2 ± 6.43 post 10.2 ± 4.23	*a) .03* *b) ns*	*n.r*	*-*	a) MTX pre 2.92 ± 1.24 post 2.95 ± 1.38 b) SLP pre 2.86 ± 1.19 post 2.81 ± 1.01	*a) ns* *b) ns*	*n.r*	*-*	*n.r*	*-*	LBR a) MTX post 32.95 (29) b) SLP post 36.3 (8)	-
Ye et al. [28]	a) 83 unilateral SLP b) 41 bilateral SLP c) 74 no surgery	*pg/mL* a) 38.3 ± 14.91 b) 41.41 ± 16.59 c) 36.49 ± 16.77	*ns*	a) 9.6 ± 1.76 b) 9.39 ± 2.12 c) 9.78 ± 1.62	*ns*	a) 7.83 ± 4.16 b) 6.98 ± 4.15 c) 8.42 ± 4.04	*ns*	a) 3.39 ± 3.03 b) 3.15 ± 2.51 c) 3.5 ± 2.6	*ns*	*n.r*	*-*	*n.r*	*-*	*n.r*	*-*	*n.r*	*-*
Odesjo et al. [30]	a) 118 unilateral SLP b) 35 unilateral salpingotomy	*n.r*	*-*	*n.r*	*-*	a) 11.69 ± 5.59 b) 11.8 ± 6.1	*ns*	*n.r*	*-*	a) 104 (88 %) b) 30 (85.7 %)	*ns*	*n.r*	*-*	a) 32 (27.1 %) b) 8 (22.9 %)	*ns*	LBR a) 21 (17.8) b) 7 (20)	*ns*

SLP, salpingectomy; n.r, not reported; n.s, not significant; ECP, ectopic pregnancy; HY, Hydrosalpinx; ECP, ectopic pregnancy; TOA, tubo-ovarian abscess; TF, tubal factor; LPS, laparoscopy; LPT, laparotomy; PTD, proximal tubal division; PTO, proximal tubal occlusion; MTX, methotrexate; LBR, live birth rate; IVF, in-vitro fertilization

### Effect on ovarian reserve markers

#### Basal FSH variations after uni/bilateral salpingectomy

A total of twelve manuscripts reported evidence regarding possible variations in FSH serum values following uni/bilateral salpingectomy [8, 10, 16, 17, 20–23, 27–29, 31]. We analyzed data from 1,569 patients. A total of 676 (43.1 %) patients underwent uni/bilateral salpingectomy, of these, 68.3 % (462 patients) underwent unilateral salpingectomy and 20 % (135 patients) bilateral salpingectomy. In a total of 79 (11.7 %) patients the technique was unspecified. Only 5.5 % (86 women) of patients underwent proximal tubal division, ligation or occlusion. We excluded from data analysis 7 (0.5 %) patients from Uyar et al. [23] because they underwent surgical procedures other than salpingectomy or tubal division.

In particular, seven papers [10, 16, 17, 20, 27, 29, 31] reported evidence from observational prospective studies evaluating FSH serum value before and after uni/bilateral salpingectomy. Six of these studies, including a total of 376 patients, did not report statistically significant differences in terms of serum FSH changes before and after surgical intervention [16, 17, 20, 27, 29, 31]. Only Gelbaya et al. [10] reported a significant increase in FSH after uni/bilateral salpingectomy in a total of 40 patients (detailed data are reported in Table 2).

The remaining five studies [8, 21–23, 28] were observational and reported evidence from the comparison of patients treated by salpingectomy with untreated controls. In particular, four manuscripts, considering a total of 290 patients treated by unilateral/bilateral salpingectomy vs. 266 controls (50 proximal tubal division/ligation/occlusion, 56 sclero-therapy, 160 patients not treated surgically) did not reveal statistically significant differences in terms of serum FSH values [8, 21–23]. Only Ye et al. [28] reported a significant increase in FSH levels in 124 patients treated vs. 74 untreated controls (Table 2).

#### Basal AMH and AFC variations after uni/bilateral salpingectomy

Concerning basal AFC and AMH values we reported evidence from a total of nine studies [13, 18, 22, 23, 25–28, 31]. Four considered only basal AFC [13, 18, 23, 27], two only AMH [25] and three considered a combination of both parameters [22, 28, 31]. We analyzed data from 1,017 patients. A total of 510 (50.2 %) underwent uni/bilateral salpingectomy; of these, 75.1 % (383 patients), underwent unilateral salpingectomy, 18.8 % (96 patients) bilateral salpingectomy and 6.1 % (31 patients) unspecified procedures. Only 23 (2.3 %) patients underwent tubal division. Three manuscripts, for a total of 237 (23.3 % of the sample) patients, defined the control group as the same cohort of patients prior to undergoing surgery [18, 27, 31]. Four manuscripts defined the control group as either untreated patients, for a total of 260 (25.6 %) or patients treated by methotrexate (MTX) for ectopic pregnancy, for a total of 202 (19.9 %) [22, 23, 25, 28]. Chan et al. [13] defined as the control group for AFC count, the counter-lateral adnexa of the same group of patients treated by unilateral salpingectomy. Findley et al. [26] established as control group patients subjected to total hysterectomy without salpingectomy. We excluded from data analysis 7 (0.7 %) patients from Uyar et al. [23] because they underwent surgical procedures other than salpingectomy or tubal division.

In three studies [18, 27, 31], the variation in basal AFC before and after unilateral/bilateral salpingectomy in the same cohort of patients was evaluated. Two, reporting evidence derived from observation of 222 patients, did not observe statistically significant differences in terms of AFC after unilateral salpingectomy [27, 31]. Only one paper evaluating a total of 15 cases of uni/bilateral salpingectomy reported a significant decrease in AFC [18]. Three manuscripts reported evidence from observational studies comparing patients surgically treated versus controls (no-treated or medically MTX) In a total of 210 patients who underwent uni/bilateral salpingectomy compared to 49 patients treated by MTX for ectopic pregnancy, 23 patients who underwent tubal division and finally 205 not untreated patients, the three Authors did not observe any statistically significant decrease in AFC [22, 23, 28]. Only Chan et al. [13] noted significant differences in a cohort of 32 patients treated by unilateral laparoscopic and laparotomy salpingectomy with the non-treated site in the same cohort assigned as controls.

Concerning AMH serum values after uni/bilateral salpingectomy a single manuscript reported evidence evaluating AMH before and after the surgical procedure in the same cohort of patients. Venturella et al. [31], in a sample of 91 and 95 patients treated by unilateral salpingectomy using standard and wide excision approach respectively, did not observe statistically significant differences in terms of decrease in AMH prior to and 3 months after surgical intervention. Three papers [22, 25, 28] reported evidence from observational studies comparing surgically treated patients versus controls. Two of the above mentioned papers compared a total of 76 cases of uni/bilateral salpingectomy versus 23 tubal ligations versus 106 non surgically treated patients and found no significant difference in terms of AMH [22, 25]. Only one paper comparing a total of 124 cases of uni/bilateral salpingectomy and 74 untreated controls found a significant decrease in AMH value in the treated group [28]. Finally, Findley et al. [26] in a cohort of 30 patients undergoing total hysterectomy (15 bilateral salpingectomies vs. 15 untreated patients) did not find significant differences in terms of AMH decrease neither within the same group, nor between the two study groups

#### Ovarian reserve markers after unilateral vs. bilateral salpingectomy

Only three papers [20, 22, 28] evaluated the impact of the type of surgical procedure by comparing unilateral vs. bilateral salpingectomy. Ni et al. [22] comparing 26 bilateral salpingectomies, 34 unilateral salpingectomies and 51 untreated controls found no significant differences, although in the bilateral salpingectomy group, a slightly inferior AMH in terms of absolute value was observed. Also Xi et al. [20] in an observational study which considered exclusively FSH levels (44 unilateral vs. 32 bilateral salpingectomy before and after surgery, and 80 untreated controls) observed no significant differences between groups. On the contrary, Ye et al. [28], analyzed 83 unilateral vs. 41 bilateral salpingectomy and 74 untreated patients, and found a significant decrease in both AMH and AFC and a significant increase in FSH in treated patients. This trend was markedly pronounced in patients treated by bilateral salpingectomy.

### Effects on ART outcomes

A total of 24 studies for a total of 3001 patients have reported data on the effects of salpingectomy on ART outcomes. Of these 1,334 were treated by uni/bilateral salpingectomy, 519 (38.9 %) patients underwent unilateral salpingectomy, 346 (25.94 %) bilateral salpingectomy and 469 (35.16 %) unspecified. The principal indication for the surgery was a previous ectopic pregnancy/ies and hydrosalpinx in 7 [1, 5, 12, 20, 27, 29, 30] and 13 studies [2–4, 6–11, 17, 18, 21, 25], respectively. In 4 studies, both patients treated for ectopic pregnancy and hydrosalpinx were included in the analysis [19, 22, 24, 28].

#### Length of stimulation

All 16 papers which analyzed the length of stimulation, defined as number of days of gonadotropin administration, found a non-statistically significant different between salpingectomy vs. non-salpingectomy patients [1–10, 12, 18–20, 28, 29].

#### E_2_ levels

Plasmatic E_2_ levels on the day of human chorionic gonadotropin (hCG) administration (for ovulation triggering) resulted nonsignificant different between salpingectomy and non-salpingectomy arms in 10 studies [1, 2, 8, 12, 17–20, 24, 28]. In only one retrospective analysis, including 40 patients treated by salpingectomy due to hydrosalpinx compared to 103 patients with tubal factor infertility (no hydrosalpinx), reported a significant reduction in E_2_ levels in the salpingectomy group [10].

#### Oocytes retrieved

A total of 21 studies described the absence of any significant difference in terms of number of oocytes retrieved between salpingectomy and non-salpingectomy patients or in pre and post-surgical procedure in the same cohort of women [2–4, 6–12, 17–22, 24, 27, 28, 30]. Lass et al. [1] compared 29 patients treated by salpingectomy for ectopic pregnancy to 73 healthy women reporting a significant reduction in oocyte yield in the ipsilateral compared to the contralateral ovary. Grynnerup et al. [25] compared a total of 16 cases of hydrosalpinx treated by salpingectomy compared to 42 patients affected by tubal factor infertility (with or without hydrosalpinx) managed conservatively. A significant reduction of oocyte yield in the treated group was observed [25].

#### Embryos obtained and/or transferred

All 11 [2, 4, 6, 7, 9–12, 20, 22, 28] and 14 [1, 3–5, 7–9, 11, 18, 22, 24, 25, 29, 30] studies which analyzed, respectively, the number of embryos obtained and of embryos transferred found no statistically significant difference between salpingectomy and non-salpingectomy patients.

#### Implantation rate

Only 6 studies assessed the implantation rate after IVF and embryo transfer. Two randomized controlled trials (RCTs) which compared patients with HY treated surgically or conservatively before IVF clearly demonstrated a significant improvement in implantation rates [4, 9]. This improvement is even more prominent in ultrasound visible hydrosalpinx [4]. One RCT and one retrospective study found no significant differences between treated and untreated women, even if surgically treated patients for hydrosalpinx showed a better implantation rate in terms of absolute value compared to untreated subjects [2, 10]. Two further retrospective studies found no significant improvement of implantation rate in patients after salpingectomy; however, in these cases, the control subjects were represented by patients with tubal factor infertility without hydrosalpinx [3, 24].

#### Clinical pregnancy rate

Clinical pregnancy rate was reported as outcome measure in 16 studies. In particular, five RCTs compared a total of 359 patients affected by hydrosalpinx treated by salpingectomy vs. 281 untreated control subjects [2, 4, 7, 9, 11]. Four of these studies reported a clear significant improvement in the pregnancy rate of treated vs. untreated subjects [4, 7, 9, 11]. The remaining RCT did not show a significant improvement in pregnancy rate, even if a better result in terms of absolute value for transfer cycle in the salpingectomy group was reported [2]. All other observational and retrospective studies, with the exception of that reported by Orvieto et al. [18], did not report differences in terms of pregnancy rate in the salpingectomy compared to the control group; however, patients in the control group were generally affected by infertility for tubal factor without hydrosalpinx or were patients who underwent a surgical or medical procedure other than salpingectomy [1, 3, 8, 10, 17, 18, 21, 22, 24, 27, 30].

#### Ongoing pregnancy/live-birth rate

Data on ongoing pregnancy and the live-birth rates were reported in 11 studies. Four RCTs compared a total 299 patients affected by hydrosalpinx treated by salpingectomy vs. 215 untreated controls [2, 4, 7, 9]. In 3 of these studies [4, 7, 9], a significant improvement in ongoing pregnancy rate/live birth rate in treated vs. untreated subjects was observed. A further RCT did not show significant improvement of pregnancy rate, even if a better result in terms of absolute value for transfer cycle after salpingectomy was reported [2]. The other available studies reported an improvement after salpingectomy group; however, they were non-randomized and poorly controlled. In fact, the controls were generally affected by infertility for tubal factor without hydrosalpinx or were patients who underwent surgical or medical procedures other than salpingectomy [3, 10, 22, 24, 27, 29, 30].

## Discussion

During the past decades many debates have been raised concerning the potential detrimental effects of hydrosalpinx (both unilateral and bilateral) on implantation and pregnancy rate after ART [32]. Two meta-analyses, published at the end of the nineties, were developed with the aim to definitively clarify this topic. The first one, analyzing more than 6,700 IVF cycles from 11 studies demonstrated a pregnancy rate of less than 49 % and a miscarriage rate 2–3 fold greater in patients with hydrosalpinx compared to controls subjects with tubal factor infertility without hydrosalpinx [odds ratio (OR 50.7), 95 % confidence interval (CI) 41.4 to 62.2) [33]. The second one, involving a total of 5,592 women (including many of the same studies used in the first meta-analysis), showed a delivery rate per cycle of 13.4 % vs. 23.4 % (OR 0.58, 95%CI 0.49 to 0.69) for hydrosalpinx group and non- hydrosalpinx group, respectively [34].

In view of the above evidence it seemed logical to propose surgery prior to ART in order to avert possible deleterious effects derived from tubal fluids and consequently increase their success rate [4]. Laparoscopic unilateral or bilateral salpingectomy has been the first and most studied surgical technique proposed. However, although several observational and retrospective studies have been published, only five RCTs (of which one published only in abstract form) concerning the potential benefit of prophylactic laparoscopic salpingectomy before ART in case of hydrosalpinx were conducted. Despite potential bias due to small sample size and lack of blind randomization, (see Table 1 for more details) data analysis in all trials showed a clear advantage in terms of implantation rate, pregnancy rate and ongoing pregnancy rate in treated patients compared to untreated controls [2, 4, 6, 7, 9, 11]. The derived data encouraged the scientific community to recommend tubal removal or tubal occlusion for hydrosalpinx prior to ART [14, 15, 32, 35]. Though these recommendations may be of use in the management of evident hydrosalpinx, controversy persists regarding the ideal management of smaller unilateral or bilateral hydrosalpinx due to the potential detrimental effects of surgery on the ovarian reserve. Analogous to the current trend in surgical management of patients affected by ovarian endometriomas [36, 37] tubal surgery before ART treatments is an ongoing debate.

With the intent of proposing a solution to the current dilemma, we analyzed several studies aimed at quantifying possible damage on ovarian reserve caused by salpingectomy. Considering data from operated vs. non-operated site, almost all studies, with the exception of Dar’ data [5], reported a significant reduction of AFC and number of oocytes retrieved after ovarian stimulation limited to the ipsilateral ovary [1, 13, 18, 25].

The pathophysiological explanation of this finding may be related to two important phenomena: surgical dissection of vascular supply (mesosalpinx vessels) and energy spread by monopolar or bipolar devices [13, 18, 31, 38]. It is important to remember that AFC is only a surrogate of ovarian reserve that should be interpreted ideally in association with basal AMH, FSH and chronological age [39–41]. Trials investigating variations in ovarian reserve determined by the combination of both AFC and biochemical assay of FSH and AMH levels seems to suggest a non-significant reduction in the cohort of treated patients compared to controls [8, 16, 17, 20–23, 25–27, 31].

This finding is likely affected by the bias of considering unilateral salpingectomy (in which the damage may happen) while the estimated ovarian reserve considers both ovaries. It is fundamental to understand the implications of bilateral vs. unilateral salpingectomy on ovarian reserve and potential response to controlled stimulation. Data regarding this last point are scarce and only three studies analyzed this topic [20, 22, 28]. In only one study [28] a significant decrease in AMH and an increase in FSH serum values in the bilateral salpingectomy group was detected, even if after ovarian stimulation there was no difference in the number of oocytes collected. Interestingly, although all other Authors did not observe significant differences, worse outcomes in terms of absolute value were reported in bilateral salpingectomy group [22, 28].

Available data is scarce and does not clarify the issue of the effect of potential additional damage caused by bilateral as opposed to unilateral salpingectomy. The lack of clear evidence does not permit speculation regarding the potential benefit of prophylactic salpingectomy in patients undergoing ART and affected by unilateral hydrosalpinx and contralateral or bilateral tubal occlusion without hydrosalpinx. Certainly, if data reported in the last trial by Venturella et al. [31] suggesting no detrimental effect on ovarian reserve in bilateral salpingectomy will be confirmed by further trials, counselling for surgery in patients affected by tubal occlusion (also in cases without hydrosalpinx) may have a rationale and may result in further improvements of ART success rates (avoiding implantation failure or even more important tubal pregnancy). Indeed, when considering as outcome measure the implantation rate, pregnancy rate and ongoing/live-birth rate after ART in patients with hydrosalpinx, data from four RCTs suggest distinct improvements in patients treated by salpingectomy [4, 6, 7, 9, 11].

Infertile women affected by hydrosalpinx are at increased risk for poor outcomes after IVF for a variety of reasons linked to chronic subclinical infections, inflammation and endometritis [42–44]. The pathophysiological explanation may be related to the possible accumulation of hydrosalpinx fluid within the endometrial cavity, which may have detrimental effects on endometrial receptivity and embryo development by a direct embryotoxic effect, mechanical washing of the blastocyst and by the dilatational effect of hydrosalpinx fluids on essential nutrients and fluids [45–52]. Moreover, although the data from these RCTs would apparently put an end to the controversy, the fact that the size and the localization (unilateral versus bilateral) of the hydrosalpinx recommend caution in the interpretation of data. The final aim of our review is non only to offer an overview of available data on this topic but to invite both scientists and clinicians in generating new data in order to finally clarify the role of salpingectomy on ovarian reserve. Indeed, just as for example, from our manuscript submission to final acceptance by the journal, 2 meta-analysis have been published in a single journal (August and October 2016) reporting opposite results and potentially generating more confusion [53, 54].

## Conclusions

From the analysis of all available literature it is possible to conclude that in the presence of tubal disease a surgical approach based on unilateral salpingectomy may be considered safe and without negative effects on ovarian reserve markers and on ovarian response to ovarian stimulation protocols. Further trials aimed at confirming both the positive effects of tubal surgery before ART and the safety of bilateral salpingectomy are necessary to definitively state when and why unilateral rather than bilateral salpingectomy are recommended in cases of bilateral tubal blockage with or without hydrosalpinx.

## Abbreviations

AMH: Anti-Mullerian hormone
ART: Assisted reproductive technologies
CI: Confidence interval
E2: Estradiol
FSH: Follicle stimulating hormone
hCG: Human chorionic gonadotropin
IVF: in vitro fertilization
MTX: Methotrexate
OR: Odd ratio
PRISMA: Preferred Reporting Items for Systematic Reviews and Meta-Analyses
RCTs: Randomized controlled trials
